# Downregulation of CD4+LAP+ and CD4+CD25+ Regulatory T Cells in Acute Coronary Syndromes

**DOI:** 10.1155/2013/764082

**Published:** 2013-12-10

**Authors:** Ying-zhong Lin, Shan-he Lu, Zheng-de Lu, Ying Huang, Ying Shi, Ling Liu, Xiao-yan Wang, Qing-wei Ji

**Affiliations:** ^1^Department of Cardiology, The People's Hospital of Guangxi Zhuang Autonomous Region, Nanning, China; ^2^Department of Ultrasound, The People's Hospital of Guangxi Zhuang Autonomous Region, Nanning, China

## Abstract

*Background*. Regulatory T (Treg) cells play a protective role in atherosclerosis prone models and are related to the onset of acute coronary syndromes (ACS, including non-ST-elevation ACS (NSTEACS) and ST-elevation acute myocardial infarction (STEAMI)). CD4+LAP+ Treg cells are a novel subset of Tregs that have been found to ameliorate atherosclerosis in ApoE^−/−^ mice, and these cells also exist in humans. The present study was designed to investigate whether CD4+LAP+ Treg cells are involved in the onset of ACS. *Methods*. The frequencies of CD4+LAP+ and CD4+CD25+ Treg cells were detected using flow cytometric analysis, and the plasma IL-10 and TGF-**β**1 levels were measured using an ELISA in 29 stable angina (SA) patients, 30 NSTEACS patients, 27 STEAMI patients, and a control group (30 cases). *Results*. The results revealed a significant decrease in the frequencies of CD4+LAP+ and CD4+CD25+ Treg cells and in the levels of IL-10 and TGF-**β**1 in patients with ACS compared with those in the SA and control groups. *Conclusions*. The decrease in the frequencies of CD4+LAP+ and CD4+CD25+ Treg cells may play a role in the onset of ACS.

## 1. Introduction

The adaptive T-cell-driven immunoinflammatory response is involved in the development of atherosclerosis and plaque destabilization that leads to the onset of acute coronary syndrome (ACS, including non-ST-elevation ACS (NSTEACS) and ST-elevation acute myocardial infarction (STEAMI)) [1–3]. Accumulating evidence has shown that CD4+ effector T (Teff) cells may accelerate atherosclerosis development [4–6]. In contrast, CD4+ regulatory T (Treg) cells play a protective role in atherosclerosis [7–10]. The best-described CD4+ Treg cells in experimental atherosclerosis are the naturally occurring CD4+CD25+ Treg cells, which have been shown to be continuously produced within the thymus. These cells play a protective role in the progression of atherosclerosis through cell-to-cell contact and, in part, the secretion of anti-inflammatory cytokines such as interleukin- (IL-) 10 and transforming growth factor-beta (TGF-*β*), and their suppressive function is dependent on the transcription factor forkhead/winged-helix transcription factor box P3 (FoxP3). Other subsets of CD4+ Treg cells, such as T-helper cell type 3 (Th3) and type 1 Treg (Tr1) cells, were also shown to attenuate atherosclerosis in apolipoprotein E-knockout mice [8–10]. However, the clinical data suggested that the activity of Treg cells was downregulated in patients with ACS [11–14]. Although the frequencies of Treg cells increased in advanced plaques compared to early plaques in humans, there was no difference between advanced plaque and early lesion, and the frequencies of Treg cells in human plaques were significantly lower than those in normal or inflammatory skin lesions, suggesting that changes take part in the smoldering chronic inflammatory process in atherosclerosis and the onset of ACS symptoms [15].

CD4+LAP+ Treg cells are a novel subset of Tregs that express latency-associated peptide (LAP) and the aminoterminal domain of TGF-*β* precursor peptide and have regulatory properties that are independent of FoxP3 [16–21]. A number of studies have shown that CD4+LAP+ Treg cells suppress the Teff cell responses and protect mice from colitis, multiple sclerosis, systemic lupus erythematosus, and diabetes via the secretion of TGF-*β* and/or IL-10 [17–21]. Recently, Sasaki et al. and our group found that CD4+LAP+ Treg cells induced by mucosal antigens efficiently suppress the immune responses of Teff cells and ameliorate atherosclerosis in ApoE^−/−^ mice [22–24]. However, the question of whether the activity of CD4+LAP+ Treg cells was regulated in ACS has not been investigated. Here, we investigated the changes in the frequencies of CD4+LAP+ and CD4+CD25+ Treg cells in patients with ACS.

## 2. Methods

### 2.1. Patients

A total number of 116 patients were enrolled in the present study, which includes four groups: (1) stable angina (SA) (17 men and 12 women, mean age 67.5 ± 8.1) and inclusion criteria are typical exertional chest discomfort that was associated with down sloping or horizontal ST-segment depression >1 mm in an exercise test; (2) NSTEACS group (21 men and 9 women, mean age 65.2 ± 10.6) and inclusion criteria are electrocardiographic (ECG) ST-segment depression or prominent T-wave inversion and/or positive biomarkers of necrosis (troponin I and Creatine Kinase MB) in the absence of ST-segment elevation and in an appropriate clinical setting (chest discomfort or anginal equivalent); (3) STEAMI group (23 men and 4 women, mean age 65.1 ± 9.8) and inclusion criteria are myocardial infarction that was confirmed by a significant increase in troponin I and Creatine Kinase MB levels and persistent ECG ST elevation; (4) the control group, which consisted of 30 subjects with normal coronary artery (17 men and 13 women, mean age 61.0 ± 9.0).

Written informed consent was obtained from each participant. The study was approved by the Ethics Committee of the People's Hospital of Guangxi Zhuang Autonomous Region, Nanning, China. Patients with valvular heart disease, thromboembolism, collagen disease, disseminated intravascular coagulation, advanced liver disease, renal failure, malignant disease, or septicemia or that were on steroid therapy were excluded from the study.

### 2.2. Blood Samples

In the NSTEACS and STEAMI groups, blood samples were obtained as soon as patients arrived. Blood samples were obtained from the other patients in the recumbent position with a 21-gauge needle with clean venipuncture of an antecubital vein in a fasting state on the following morning of the admission day. The samples were collected into sodium heparin vacutainers (Becton Dickinson). The peripheral blood mononuclear cells (PBMCs) were prepared by Ficoll density gradient for flow cytometric analysis. The plasma obtained after centrifugation was stored at −80°C until further use.

### 2.3. Flow Cytometric Analysis

The cells were stained with surface markers as anti-LAP-APC (RD Systems), followed by anti-CD4-FITC (eBioscience) and anti-CD25-PE (eBioscience). The isotype controls were given to enable correct compensation and confirm antibody specificity. The stained cells were analyzed by flow cytometric analysis using a FACScan cytometer equipped with CellQuest software (BD Bioscience Pharmingen).

### 2.4. ELISA Detection of the Levels of TGF-*β*1 and IL-10

The levels of TGF-*β*1 and IL-10 were measured by enzyme-linked immunosorbent assay (ELISA) following the manufacturer's instructions (WestTang Biotech, Shanghai, China). The minimal detectable concentrations were 15 pg/mL for TGF-*β*1 and IL-10. Intraassay and inter-assay coefficients of variation for all ELISA were <5% and <10%, respectively. All samples were measured in duplicate.

### 2.5. Gensini Score

The severity of coronary stenosis in patients was estimated with a Gensini coronary score following coronary angiography. The Gensini score was computed by assigning a severity score to each coronary stenosis according to the degree of luminal narrowing and its geographic importance. The reduction in the lumen diameter and the roentgenographic appearance of concentric lesions and eccentric plaques were evaluated (reductions of 25%, 50%, 75%, 90%, and 99% and complete occlusion were assigned Gensini scores of 1, 2, 4, 8, 16, and 32, resp.). The score was then multiplied by a factor that incorporates the importance of the lesion's position in the coronary arterial tree as follows: 5 for the left main coronary artery; 2.5 for the proximal left anterior descending coronary artery (LAD) or the left circumflex artery (LCX); 1.5 for the mid-LAD; and 1 for the distal LAD, the right coronary artery, or the middistal LCX.

## 3. Statistical Analysis

All of the data were given as the mean ± SD. When comparing only 2 groups, Student's *t*-test was used. For comparisons involving 3 or more groups, one-way ANOVA followed by Newman-Keuls post hoc test was used. Spearman's correlation was used to calculate the correlations between two continuous variables. In all tests a value of *P* < 0.05 was considered to be statistically significant.

## 4. Results

There was no significant difference in age, gender, history of hypertension, diabetes, or tobacco use in these four groups. The left ventricular ejection fraction (LVEF) in the STEAMI group was lower than that of the control group, whereas the levels of C-reactive protein (CRP), the Gensini score, and the left ventricular end-diastolic dimension (LVEDD) were significantly higher in the STEAMI group than in the control group. The other parameters including lipid and lipoprotein fractions, fasting glucose, and prehospital medications are listed in Table 1.

**Figure 1:**
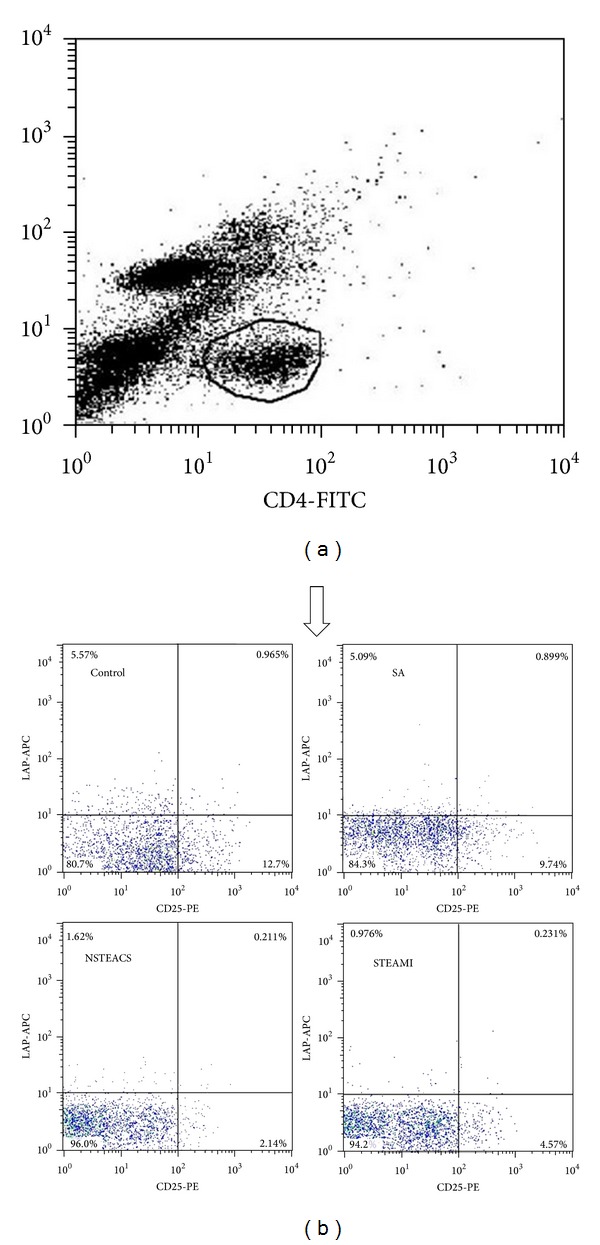
The frequencies of the CD4+LAP+ and CD4+CD25+ T cells in each group. (a) CD4+ T cells were gated by flow cytometry. (b) Representation of surface staining of the CD4+LAP+ and CD4+CD25+ T cells from each group.

**Figure 2:**
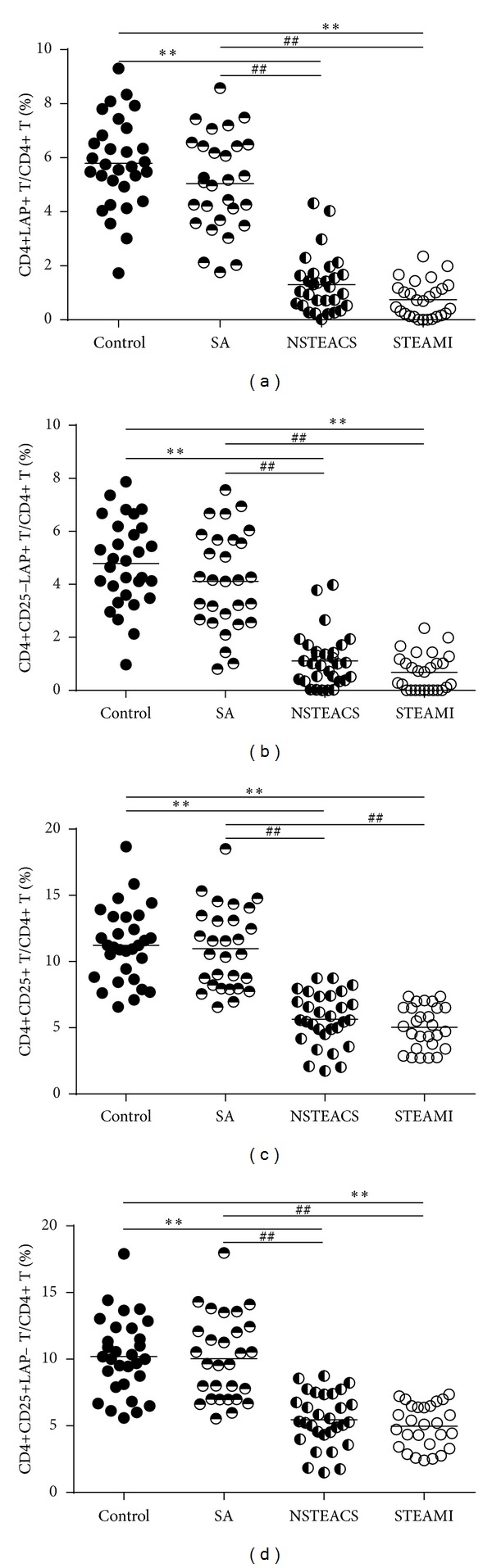
Circulating CD4+LAP+ and CD4+CD25+ T cells decreased in ACS. (a) The frequencies of CD4+LAP+ T cells were markedly lower in patients with STEAMI and NSTEACS than those in the control and SA groups. (b) The frequencies of CD4+CD25−LAP+ T cells were markedly lower in patients with STEAMI and NSTEACS than those in the control and SA groups. (c) The frequencies of CD4+CD25+ T cells were markedly lower in patients with STEAMI and NSTEACS than those in the control and SA groups. (d) The frequencies of CD4+CD25+LAP− T cells were markedly lower in patients with STEAMI and NSTEACS than those in the control and SA groups. **P < 0.01 versus control and ^##^ *P* < 0.01 versus SA group.

As shown in Table 2 and Figures 1 and 2, the frequencies of the CD4+LAP+ T cells, the CD4+CD25−LAP+ T cells, the CD4+CD25+ T cells, and the CD4+CD25+LAP− T cells were significantly decreased in patients with STEAMI and NSTEACS than those in the SA and control groups, but no obvious difference was found between the SA group and the control group. In total, 86 CAD patients were divided into the single-, double-, and triple-vessel disease groups according to the angiographic results and there were no differences in the frequencies of CD4+LAP+ T cells, CD4+CD25−LAP+ T cells, CD4+CD25+ T cells, and CD4+CD25+LAP− T cells among the three groups (see Table 2). Furthermore, 116 patients were divided into a hypertension group (67 cases) and a normotension group (49 cases) or a diabetes group (20 cases) and a nondiabetes group (96 cases). The results showed that there were no significant differences in the frequencies of CD4+LAP+ T cells, CD4+CD25−LAP+ T cells, CD4+CD25+ T cells, and CD4+CD25+LAP− T cells between the hypertension group and the normotension group or between the diabetes group and the nondiabetes group (see Table 3). In addition, there were no significant differences in the frequencies of CD4+LAP+ T cells, CD4+CD25−LAP+ T cells, CD4+CD25+ T cells, and CD4+CD25+LAP− T cells based on sex, smoking, and drug treatment (see Tables 3 and 4).

**Figure 3:**
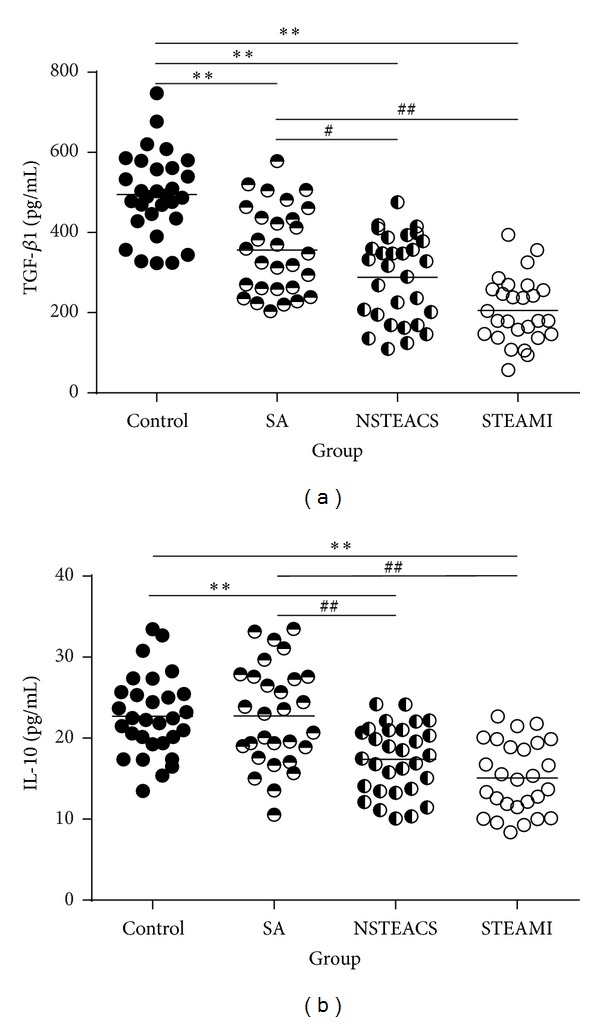
The plasma TGF-*β*1 and IL-10 concentrations analysis. (a) The plasma TGF-*β*1 levels in patients with STEAMI, NSTEACS, and SA were significantly decreased compared with those of the control group, and the plasma TGF-*β*1 levels in patients with STEAMI and NSTEACS were significantly decreased compared with those of the SA group. (b) The plasma IL-10 levels in patients with STEAMI, NSTEACS, and SA were significantly decreased compared with those of the control and SA groups. **P < 0.01 versus control, ^#^ *P* < 0.05 versus SA group, and ^##^ *P* < 0.01 versus SA group.

**Figure 4:**
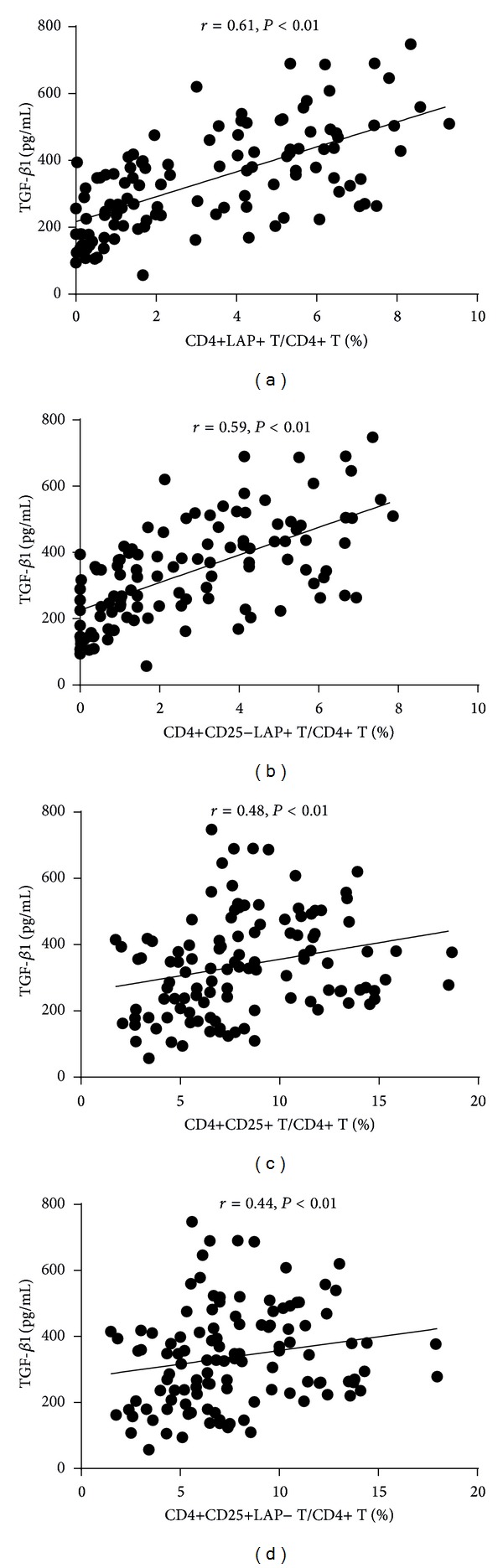
The plasma TGF-*β*1 levels showed positive correlation with the frequencies of CD4+LAP+ T cells, CD4+CD25−LAP+ T cells, CD4+CD25+ T cells, and CD4+CD25+ LAP− T cells.

**Figure 5:**
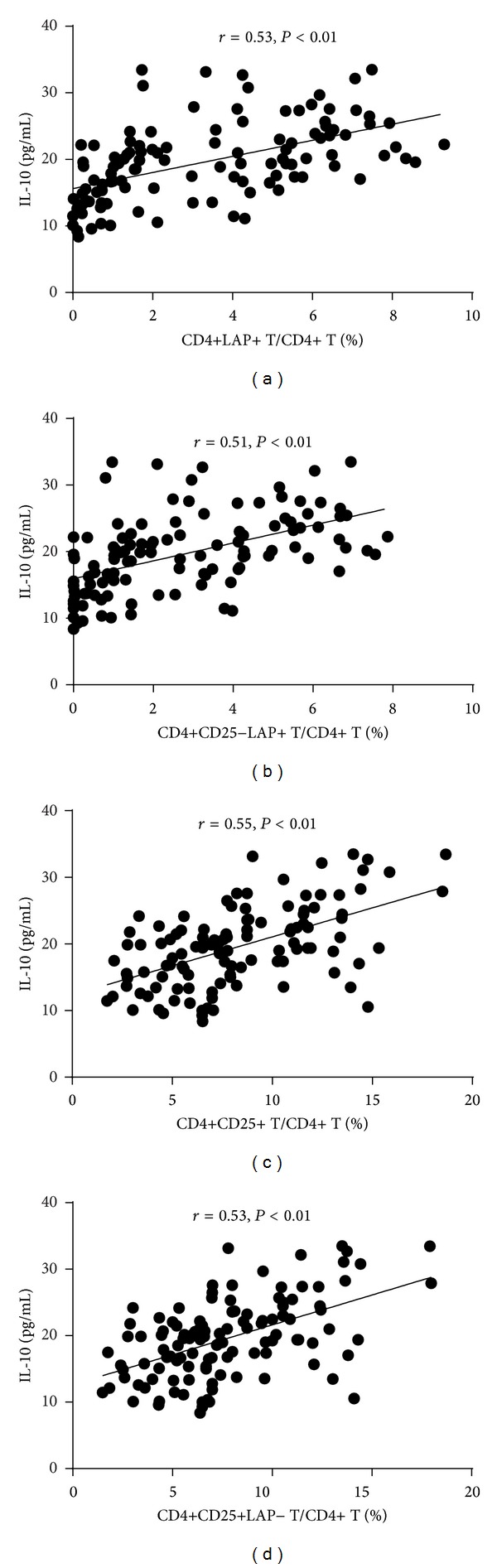
The IL-10 levels showed positive correlation with the frequencies of CD4+LAP+ T cells, CD4+CD25−LAP+ T cells, CD4+CD25+ T cells, and CD4+CD25+LAP− T cells.

As shown in Figure 3, the plasma TGF-*β*1 and IL-10 levels in patients with STEAMI (205.72 ± 82.05 pg/mL and 15.08 ± 5.93 pg/mL, resp.) and NSTEACS (288.51 ± 105.92 pg/mL and 17.38 ± 4.17 pg/mL, resp.) were significantly decreased compared with those of the control group (491.53 ± 126.59 pg/mL and 22.71 ± 4.90 pg/mL, resp.) and the SA group (356.43 ± 112.27 pg/mL and 22.76 ± 6.16 pg/mL, resp.). In addition, the plasma TGF-*β*1 and IL-10 levels showed positive correlation with the frequencies of CD4+LAP+ T cells, CD4+CD25−LAP+ T cells, CD4+CD25+ T cells, and CD4+CD25+LAP− T cells (Figures 4 and 5).

We assessed whether the frequencies of CD4+LAP+ and CD4+CD25+ Treg cells were associated with age, lipid and lipoprotein fractions, fasting glucose, CRP, and the Gensini score which used to quantify the severity of coronary artery stenosis in patients with coronary artery disease (CAD). As shown in Table 5, the frequencies of CD4+LAP+ and CD4+CD25+ Treg cells were positively correlated with total cholesterol (TC), low-density lipoprotein cholesterol (LDL-C), and CRP, and there was no correlation with age, total triglycerides (TG), high-density lipoprotein cholesterol (HDL-C), fasting glucose, and the Gensini score. Because LVEF, LVEDD, and the GRACE score are related to the prognosis in patients with ACS, we analyzed the correlation between Tregs and these parameters in ACS patients. As shown in Table 5, the frequencies of CD4+LAP+ and CD4+CD25+ Treg cells were positively correlated with LVEF but negatively correlated with LVEDD in ACS patients. The results showed that there was no correlation between the frequencies of CD4+LAP+ and CD4+CD25+ Treg cells and GRACE score in patients with ACS.

## 5. Discussion

Oida et al. first identified a new regulatory CD4+ T cell phenotype that is CD25− and LAP+, and they found that CD4+LAP+ CD25− T cells could effectively ameliorate CD4+CD45RB^high^-induced colitis by a TGF-*β*-dependent mechanism [17]. In addition to the production of TGF-*β*, CD4+LAP+ Treg cells were found to exert their suppressive function through cell-to-cell contact and the secretion of IL-10 [16–21]. CD4+LAP+ Treg cells induced by mucosal antigens, such as anti-CD3 antibody, significantly suppressed colitis, multiple sclerosis, systemic lupus erythematosus, and diabetes [17–21]. Mucosal antigens induced both CD4+LAP+ and CD4+ FoxP3+ Treg cells, and the majority of CD4+LAP+ Treg cells did not express FoxP3 and, conversely, the majority of CD4+ FoxP3+ Treg cells did not express LAP [21]. In addition, transfer of the sorted CD4+LAP+ Treg cells was confirmed to inhibit the Teff cell responses and attenuate asthmatic lung inflammation in vivo. These results indicated that CD4+LAP+ Treg cells may be mainly an inducible subset of Treg cells, and their suppressive function is independent of FoxP3, whereas CD4+CD25+ Treg cells act as the natural Treg cells and FoxP3 is critical for their suppressive function. Recently, the role of CD4+LAP+ Treg cells in atherosclerosis has been extensively investigated [22–24]. Sasaki et al. [22] first found that oral anti-CD3 antibody administration could induce CD4+LAP+ Treg cells, and therefore these cells suppress the Teff cell response and attenuate atherosclerosis in ApoE^−/−^ mice. We also found that CD4+LAP+ Treg cells induced by nasal ox-LDL produce an amount of TGF-*β* and may play a protective role in atherosclerosis. Because we did not sort CD4+LAP+ Treg cells and CD4+CD25+FoxP3+ Treg cells to transfer into ApoE^−/−^ mice, respectively, and CD4+CD25+ FoxP3+ Treg cells were also induced by nasal ox-LDL, it is difficult to confirm which type of Treg cells is the most important in this process. In our opinion, both CD4+LAP+ and CD4+CD25+FoxP3+ Treg cells play an important role, and the other cells such as macrophages may also take part in this process. More recently, another study from the Huang group showed that oral FTY720 induced CD4+LAP+ Treg cells and inhibited early lesion in ApoE^−/−^ mice [24].

Notably, CD4+LAP+ Treg cells are found in human and these cells do not express FoxP3, but they exert suppressive function [16]. Because Th3 cells, a subset of Treg cells that mediate their suppressive activities via the release of TGF-*β* [25, 26], and CD4+CD25+ Treg cells were downregulated in ACS [11–14], we wanted to investigate whether CD4+LAP+ Treg cell activity is altered in ACS.

Similar to other studies, the present study revealed that the frequencies of CD4+CD25+ Treg cells were significantly decreased in patients with ACS. Furthermore, we first reported that the frequencies of CD4+LAP+ Treg cells were downregulated in patients with ACS compared with those in the SA and control groups, suggesting that a decrease in the frequencies of CD4+LAP+ and CD4+CD25+ Treg cells may be related to the plaque destabilization and the onset of ACS. In addition, the frequencies of double positive cells (LAP/CD25+) are very small and even undetectable in some patients with ACS. It is not controversial that like CAD, hypertension and diabetes are also chronic inflammatory diseases. Treg cells play a protective role in models of both hypertension and diabetes, and the function of CD4+CD25+ Treg cells was impaired in hypertension and diabetes [27–30]. We therefore investigated whether this change could have been observed in the present study. However, there were no differences between the hypertension group and the normotension group and between the diabetes group and the nondiabetes group, indicating that the inflammatory response is stronger in ACS than in hypertension and diabetes.

Accumulating evidence indicated that the activity of Treg cells, including the number and the functional suppressive properties, may be associated with lipoprotein fractions. The serum levels of ox-LDL were negatively correlated with the frequency of Treg cells, and ox-LDL is found to significantly reduce both the number and the functional suppressive properties of CD4+CD25+ Treg cells in vitro [11, 31]. Another study from Ammirati found that the frequency of Treg cells was not correlated with TC, TG, and LDL-C, but it was significantly negatively correlated with HDL-C [13]. Evidence from a cohort study did not find the correlation between lipoprotein fractions (including TG, LDL-C, HDL-C, and LDL/HDL ratio) and the frequency of Treg cells [32]. In the present study, we did not find the significant correlation between Treg cells and HDL-C in patients with coronary artery disease. In contrast, the significant correlation between CD4+LAP+ and CD4+CD25+ Treg cells and TC and LDL-C was observed in the present study. However, the reason for this discrepancy remains unclear. The difference in subjects enrolled may at least in part contribute to this discrepancy. For example, the correlations were assessed in CAD patients in the present study, whereas normal subjects were used in the cohort study.

There was no difference in the frequency of CD4+LAP+ and CD4+CD25+ Treg cells among single-, double-, or triple-vessel diseases, and no correlation was found between the frequency of CD4+LAP+ and CD4+CD25+ Treg cells and the Gensini score, which is a marker of the severity of coronary artery stenosis. These results are in line with Ammirati's study [13], in which the frequency of CD4+CD25^high^CD127^low^ Treg cells was unrelated to the degree of atherosclerosis in the carotid and coronary arteries. Because lower LVEF and an increase in LVEDD are associated with myocardial contractile depression and myocardial remodeling and lower LVEF, higher LVEDD, and a higher GRACE risk score result in a worse prognosis for ACS patients, the correlation between CD4+LAP+ and CD4+CD25+ Treg cells and LVEF, LVEDD, and the GRACE risk score were assessed. We found that the frequency of CD4+LAP+ and CD4+CD25+ Treg cells was positively correlated with LVEF, negatively correlated with LVEDD, and not correlated with the GRACE risk score in ACS patients. Recently, the baseline numbers of Tregs in 700 randomly selected subjects in the cardiovascular cohort of the Malmo Diet and Cancer Study were measured using flow cytometry, and the relationships of the baseline Treg counts and the first event of cardiovascular disease were analyzed after over a ten-year followup [32]. The results showed that low levels of CD4+FoxP3+ Treg cells but not CD4+CD25+FoxP3+ Treg cells were associated with an increased risk for myocardial infraction, and neither CD4+ FoxP3+ Treg cells nor CD4+CD25+FoxP3+ Treg cells were associated with stroke. However, a prospective cohort study on the frequency of Treg cells and prognosis in ACS patients is still lacking, although many clinical investigations have found that the frequencies of Treg cells were decreased in ACS patients.

There are some limitations in the present study. First, because FoxP3 is not critical for CD4+LAP+ Treg cells, we did not measure FoxP3 in this study. Second, the suppressive function of CD4+LAP+ Treg cells was not examined and a small fraction of the population was enrolled in the present study. Finally, although we first found that the frequencies of CD4+LAP+ Treg cells were downregulated in ACS, we failed to followup with these patients, and therefore whether the lower levels of those cells will be associated with a worse prognosis in ACS remain uncertain.

In conclusion, CD4+LAP+ Treg cells were downregulated in patients with ACS, suggesting a potential role for CD4+LAP+ Treg cells in the onset of ACS. Because lower levels of CD4+ FoxP3+ Treg cells have been found to relate to an increased risk for myocardial infarction in subjects with no coronary artery disease in a prospective cohort study [32], the exact meaning of that decrease in both CD4+LAP+ and CD4+CD25+ Treg cells in ACS should be further investigated.

## Figures and Tables

**Table 1:** Clinical characteristics of patients.

Characteristics	Control (*n* = 30)	SA (*n* = 29)	NSTEACS (*n* = 30)	STEAMI (*n* = 27)
Age (years)	61.0 ± 9.0	67.5 ± 8.1	65.2 ± 10.6	65.1 ± 9.8
Sex (male/female)	17/13	17/12	21/9	23/4
Hypertension, *n* (%)	16 (53.3)	20 (69.0)	19 (63.3)	12 (44.4)
Diabetes, *n* (%)	4 (13.3)	6 (20.7)	7 (23.3)	3 (11.1)
Tobacco, *n* (%)	5 (16.7)	10 (34.5)	5 (16.7)	7 (25.9)
TC (mmol/L)	3.95 ± 0.93	4.03 ± 0.58	4.24 ± 1.13	4.55 ± 0.82
TG (mmol/L)	1.44 ± 0.73	1.61 ± 0.71	1.98 ± 1.48	1.42 ± 1.08
LDL-C (mmol/L)	2.25 ± 0.61	2.39 ± 0.68	2.50 ± 0.86	2.67 ± 0.72
HDL-C (mmol/L)	1.26 ± 0.37	1.09 ± 0.28	1.31 ± 0.75	1.04 ± 0.41
GLU (mmol/L)	5.15 ± 1.46	5.89 ± 2.72	5.75 ± 2.36	6.07 ± 2.54
CRP (mg/L)	1.95 ± 1.63	2.62 ± 2.32	4.91 ± 1.92*	5.93 ± 2.01*
LVEF (%)	66.00 ± 4.41	62.55 ± 6.12	59.23 ± 11.16	43.41 ± 8.60*
LVEDD (mm)	46.33 ± 3.53	47.41 ± 3.74	49.87 ± 5.18	54.04 ± 4.24
Gensini score	0	26.66 ± 21.77*	40.35 ± 25.00*	58.81 ± 29.64*
GRACE score	—	—	106.07 ± 27.74	160.93 ± 22.58

Medications, *n* (%)				
Aspirin	5 (16.7)	12 (41.4)	12 (40.0)	5 (18.5)
*β*-Blockers	4 (13.3)	6 (20.7)	7 (23.3)	4 (14.8)
ACEI/ARB	5 (16.7)	9 (31.0)	8 (26.7)	5 (18.5)
CCB	10 (33.3)	12 (41.4)	13 (43.3)	7 (25.9)
Nitrates	3 (10.0)	10 (34.5)	8 (26.7)	5 (18.5)
Statins	2 (6.7)	7 (24.1)	6 (20.0)	3 (11.1)

The data are given as the mean ± SD or number of patients. SA: stable angina; NSTEACS: non-ST-elevation acute coronary syndrome; STEAMI: ST-elevation acute myocardial infarction; TC: total cholesterol; TG: total triglycerides; LDL-C: low-density lipoprotein cholesterol; HDL-C: high-density lipoprotein cholesterol; GLU: fasting glucose; CRP: C-reactive protein; LVEF: left ventricular ejection fraction; LVEDD: left ventricular end-diastolic dimension; ACEI: angiotensin-converting enzyme inhibitor; ARB: angiotensin receptor blocker; CCB: calcium channel blocker.

*P < 0.05 versus control.

**Table 2:** The frequencies of circulating Treg cells in coronary artery disease.

	CD4+LAP+ T cells	CD4+CD25−LAP+ T cells	CD4+CD25+ T cells	CD4+CD25+LAP− T cells
Control	5.80 ± 1.67	4.79 ± 1.64	11.22 ± 2.78	10.21 ± 2.86
SA	5.04 ± 1.78	4.11 ± 1.84	10.97 ± 3.02	10.05 ± 3.10
NSTEACS	1.30 ± 1.05^∗∗,##^	1.11 ± 1.01^∗∗,##^	5.65 ± 1.98^∗∗,##^	5.46 ± 2.02^∗∗,##^
STEAMI	0.75 ± 0.67^∗∗,##^	0.68 ± 0.70^∗∗,##^	5.03 ± 1.60^∗∗,##^	4.97 ± 1.63^∗∗,##^
Diseased vessel				
Single-vessel	2.91 ± 2.46	2.40 ± 2.14	7.37 ± 3.84	6.86 ± 3.60
Double-vessel	2.17 ± 2.24	1.79 ± 1.94	7.11 ± 3.40	6.74 ± 3.14
Triple-vessel	2.25 ± 2.21	1.90 ± 1.95	7.33 ± 3.48	6.98 ± 3.26

The data are given as the mean ± SD. **P < 0.01 versus control and ^##^ *P* < 0.01 versus SA.

**Table 3:** The frequencies of circulating Treg cells in traditional risk factors.

	Number	CD4+LAP+ T cells	CD4+CD25−LAP+ T cells	CD4+CD25+ T cells	CD4+CD25+LAP− T cells
Male	78	3.01 ± 2.46	2.48 ± 2.09	7.79 ± 3.70	7.26 ± 3.44
Female	38	3.73 ± 2.83	3.13 ± 2.51	9.15 ± 3.73	8.54 ± 3.46
Hypertension	67	3.48 ± 2.53	2.91 ± 2.21	8.45 ± 3.89	7.87 ± 3.62
Normotension	49	2.98 ± 2.71	2.45 ± 2.33	8.04 ± 3.58	7.51 ± 3.32
Diabetes	20	2.95 ± 2.37	2.47 ± 2.07	8.50 ± 3.74	8.03 ± 3.48
Nondiabetes	96	3.34 ± 2.66	2.76 ± 2.31	8.23 ± 3.77	7.66 ± 3.50
Smoking	25	3.13 ± 2.67	2.61 ± 2.29	7.37 ± 3.36	6.85 ± 3.05
Nonsmoking	91	3.31 ± 2.61	2.74 ± 2.27	8.52 ± 3.83	7.95 ± 3.57

The data are given as the mean ± SD.

**Table 4:** The frequencies of circulating Treg cells according to medication.

	CD4+LAP+ T cells	CD4+CD25−LAP+ T cells	CD4+CD25+ T cells	CD4+CD25+LAP− T cells
Aspirin (yes)	3.42 ± 2.61	2.88 ± 2.23	7.95 ± 3.76	7.40 ± 3.50
Aspirin (no)	3.21 ± 2.62	2.64 ± 2.29	8.42 ± 3.77	7.85 ± 3.49
*β*-Blockers (yes)	3.98 ± 2.88	3.42 ± 2.50	7.79 ± 3.22	7.23 ± 2.99
*β*-Blockers (no)	3.11 ± 2.53	2.56 ± 2.19	8.39 ± 3.87	7.83 ± 3.59
ACEI/ARB (yes)	3.55 ± 2.37	3.00 ± 2.08	7.64 ± 3.55	7.09 ± 3.28
ACEI/ARB (no)	3.18 ± 2.68	2.63 ± 2.32	8.47 ± 3.81	7.91 ± 3.54
CCB (yes)	3.67 ± 2.48	3.11 ± 2.18	7.98 ± 3.80	7.42 ± 3.51
CCB (no)	3.04 ± 2.67	2.49 ± 2.29	8.45 ± 3.75	7.89 ± 3.48
Nitrates (yes)	3.47 ± 2.38	2.92 ± 2.05	7.60 ± 3.43	7.05 ± 3.17
Nitrates (no)	3.21 ± 2.68	2.65 ± 2.33	8.48 ± 3.84	7.92 ± 3.57
Statins (yes)	3.47 ± 2.33	2.90 ± 2.00	7.41 ± 3.17	6.83 ± 2.92
Statins (no)	3.23 ± 2.61	2.68 ± 2.32	8.44 ± 3.85	7.88 ± 3.57

The data are given as the mean ± SD.

**Table 5:** Spearman's correlation analysis.

	CD4+LAP+ T cells	CD4+CD25−LAP+ T cells	CD4+CD25+ T cells	CD4+CD25+LAP− T cells
Age (years)	0.03	0.03	0.05	0.07
TC (mmol/L)	−0.29**	−0.30**	−0.22*	−0.21*
TG (mmol/L)	−0.06	−0.06	−0.08	−0.08
LDL-C (mmol/L)	−0.24*	−0.24*	−0.19*	−0.19*
HDL-C (mmol/L)	0.00	0.00	0.01	0.02
GLU (mmol/L)	−0.12	−0.10	0.03	0.06
CRP (mg/L)	−0.38**	−0.35**	−0.34**	−0.33**
Gensini score	−0.17	−0.15	−0.16	−0.12
LVEF (%)	0.33**	0.28**	0.43**	0.41**
LVEDD (mm)	−0.29**	−0.25*	−0.38**	−0.36**
GRACE score	−0.21	−0.18	0.09	0.11

*P < 0.05 and **P < 0.01.

## References

[1] Libby P, Ridker PM, Hansson GK (2009). Inflammation in Atherosclerosis. From pathophysiology to practice. *Journal of the American College of Cardiology*.

[2] Andersson J, Libby P, Hansson GK (2010). Adaptive immunity and atherosclerosis. *Clinical Immunology*.

[3] Lahoute C, Herbin O, Mallat Z, Tedgui A (2011). Adaptive immunity in atherosclerosis: mechanisms and future therapeutic targets. *Nature Reviews*.

[4] Frostegård J, Ulfgren AK, Nyberg P (1999). Cytokine expression in advanced human atherosclerotic plaques: dominance of pro-inflammatory (Th1) and macrophage-stimulating cytokines. *Atherosclerosis*.

[5] Methe H, Brunner S, Wiegand D, Nabauer M, Koglin J, Edelman ER (2005). Enhanced T-helper-1 lymphocyte activation patterns in acute coronary syndromes. *Journal of the American College of Cardiology*.

[6] Eid RE, Rao DA, Zhou J (2009). Interleukin-17 and interferon-*γ* Are produced concomitantly by human coronary artery-infiltrating T cells and act synergistically on vascular smooth muscle cells. *Circulation*.

[7] Ait-Oufella H, Salomon BL, Potteaux S (2006). Natural regulatory T cells control the development of atherosclerosis in mice. *Nature Medicine*.

[8] Mallat Z, Gojova A, Brun V (2003). Induction of a regulatory T cell type I response reduces the development of atherosclerosis in apolipoprotein E-knockout mice. *Circulation*.

[9] Zhou X, Johnston TP, Johansson D (2009). Hypercholesterolemia leads to elevated TGF-*β*1 activity and T helper 3-dependent autoimmune responses in atherosclerotic mice. *Atherosclerosis*.

[10] Klingenberg R, Lebens M, Hermansson A (2010). Intranasal immunization with an apolipoprotein B-100 fusion protein induces antigen-specific regulatory T cells and reduces atherosclerosis. *Arteriosclerosis, Thrombosis, and Vascular Biology*.

[11] Mor A, Luboshits G, Planer D, Keren G, George J (2006). Altered status of CD4+CD25+ regulatory T cells in patients with acute coronary syndromes. *European Heart Journal*.

[12] Zhang WC, Wang J, Shu YW (2012). Impaired thymic export and increased apoptosis account for regulatory T cell defects in patients with non-ST segment elevation acute coronary syndrome. *The Journal of Biological Chemistry*.

[13] Ammirati E, Cianflone D, Banfi M (2010). Circulating CD4+CD25hiCD127lo regulatory T-cell levels do not reflect the extent or severity of carotid and coronary atherosclerosis. *Arteriosclerosis, Thrombosis, and Vascular Biology*.

[14] Ji QW, Guo M, Zheng JS (2009). Downregulation of T helper cell type 3 in patients with acute coronary syndrome. *Archives of Medical Research*.

[15] de Boer OJ, van der Meer JJ, Teeling P, van der Loos CM, van der Wal AC (2007). Low numbers of FOXP3 positive regulatory T cells are present in all developmental stages of human atherosclerotic lesions. *PloS ONE*.

[16] Gandhi R, Farez MF, Wang Y, Kozoriz D, Quintana FJ, Weiner HL (2010). Cutting edge: human latency-associated peptide+ T cells: a novel regulatory T cell subset. *Journal of Immunology*.

[17] Oida T, Zhang X, Goto M (2003). CD4+CD25- T cells that express latency-associated peptide on the surface suppress CD4+CD45RBhigh-induced colitis by a TGF-*β*-dependent mechanism. *Journal of Immunology*.

[18] Ochi H, Abraham M, Ishikawa H (2006). Oral CD3-specific antibody suppresses autoimmune encephalomyelitis by inducing CD4+CD25-LAP+ T cells. *Nature Medicine*.

[19] Ishikawa H, Ochi H, Chen ML, Frenkel D, Maron R, Weiner HL (2007). Inhibition of autoimmune diabetes by oral administration of anti-CD3 monoclonal antibody. *Diabetes*.

[20] Wu HY, Center EM, Tsokos GC, Weiner HL (2009). Suppression of murine SLE by oral anti-CD3: inducible CD4+CD25-LAP+ regulatory T cells control the expansion of IL-17+ follicular helper T cells. *Lupus*.

[21] Duan W, So T, Mehta AK, Choi H, Croft M (2011). Inducible CD4+LAP+Foxp3- regulatory T cells suppress allergic inflammation. *Journal of Immunology*.

[22] Sasaki N, Yamashita T, Takeda M (2009). Oral anti-CD3 antibody treatment induces regulatory t cells and inhibits the development of atherosclerosis in mice. *Circulation*.

[23] Zhong Y, Wang X, Ji Q (2012). CD4+LAP+ and CD4+CD25+Foxp3+ regulatory T cells induced by nasal oxidized low-density lipoprotein suppress effector T cells response and attenuate atherosclerosis in ApoE-/- mice. *Journal of Clinical Immunology*.

[24] Huang K, Li SQ, Wang WJ (2012). Oral FTY720 administration induces immune tolerance and inhibits earlydevelopment of atherosclerosis in apolipoprotein E-deficient mice. *International Journal of Immunopathology and Pharmacology*.

[25] Fukaura H, Kent SC, Pietrusewicz MJ, Khoury SJ, Weiner HL, Hafler DA (1996). Induction of circulating myelin basic protein and proteolipid protein- specific transforming growth factor-*β*1-secreting Th3 T cells by oral administration of myelin in multiple sclerosis patients. *The Journal of Clinical Investigation*.

[26] Weiner HL (2001). Induction and mechanism of action of transforming growth factor-*β*-secreting Th3 regulatory cells. *Immunological Reviews*.

[27] Barhoumi T, Kasal DA, Li MW (2011). T regulatory lymphocytes prevent angiotensin II-induced hypertension and vascular injury. *Hypertension*.

[28] Viel EC, Lemarié CA, Benkirane K, Paradis P, Schiffrin EL (2010). Immune regulation and vascular inflammation in genetic hypertension. *American Journal of Physiology—Heart and Circulatory Physiology*.

[29] Eller K, Kirsch A, Wolf AM (2011). Potential role of regulatory T cells in reversing obesity-linked insulin resistance and diabetic nephropathy. *Diabetes*.

[30] Zeng C, Shi X, Zhang B (2012). The imbalance of Th17/Th1/Tregs in patients with type 2 diabetes: relationship with metabolic factors and complications. *Journal of Molecular Medicine*.

[31] Li Q, Wang Y, Chen K (2010). The role of oxidized low-density lipoprotein in breaking peripheral Th17/Treg balance in patients with acute coronary syndrome. *Biochemical and Biophysical Research Communications*.

[32] Wigren M, Bjorkbacka H, Andersson L (2012). Low levels of circulating CD4+FoxP3+ T cells are associated with an increased risk for development of myocardial infarction but not for stroke. *Arteriosclerosis, Thrombosis, and Vascular Biology*.

